# Musculoskeletal symptoms and quality of life among workers at an intensive care unit in Teresina, Piauí, Brazil

**DOI:** 10.5327/Z1679443520190381

**Published:** 2019-12-01

**Authors:** Brena Costa de Oliveira, Valéria Monteiro Beserra da Silva, Letícia Graziela Lopes França Sousa, Iara Sayuri Shimizu

**Affiliations:** 1 Universidade Estadual do Piauí-Teresina, Piauí, Brazil. Universidade Estadual do Piauí Universidade Estadual do Piauí Brazil

**Keywords:** quality of life, occupational health, intensive care units, musculoskeletal pain

## Abstract

**Background:**

Having more than job and physical exhaustion derived from the job demands considerably overload workers and impair their quality of life (QoL). These factors might interfere with the health of this population of workers reflecting as work-related musculoskeletal disorders.

**Aim:**

To investigate the correlation between musculoskeletal symptoms and QoL among workers at the intensive care unit of a public hospital in Teresina, Piauí, Brazil.

**Methods:**

Cross-sectional quantitative, observational and analytical study performed with 30 workers at a public hospital ICU. WHOQOL-BREF, the Nordic Musculoskeletal Questionnaire and a sample profile instrument were administered by the investigators during the working hours.

**Results:**

86.7% of the sample reported pain in at least one body side, particularly in the knees in the past 7 seven days (23.3%) and in the lower back in the past 12 months (50%). The participants’ QoL was reasonable. We found significant moderate correlation between pain and WHOQOL domains physical health (p=0.10) and social relationships (p=0.011) but no correlation with the participants general characteristics.

**Conclusion:**

Occurrence of musculoskeletal symptoms might interfere with the QoL, social life and performance at work of ICU workers.

## RESUMO |

**Introdução:**

O acúmulo de vínculos empregatícios e o desgaste físico causado pelas exigências do trabalho provocam grande sobrecarga nos profissionais, levando ao comprometimento de sua qualidade de vida (QV). Esses fatores podem interferir na saúde desses profissionais, refletindo-se em distúrbios osteomusculares relacionados ao trabalho.

**Objetivos:**

Verificar e correlacionar a presença de sintomas osteomusculares e a qualidade de vida dos profissionais de saúde da unidade de terapia intensiva (UTI) de um hospital público de Teresina.

**Métodos:**

Estudo de campo, quantitativo, de caráter observacional, transversal e analítico, realizado com 30 profissionais da UTI de um hospital público, por meio de questionários que foram aplicados pessoalmente e durante o horário de trabalho dos participantes, sendo eles: o questionário de qualidade de vida (WHOQOL-BREF), o *Nordic musculoskeletal questionnaire* (NMQ) e um questionário sobre o perfil dos profissionais.

**Resultados:**

Dos profissionais, 86,7% sentiam dor em pelo menos uma parte do corpo, de modo que a área mais mencionada nos últimos sete dias foi o joelho (23,3%) e nos últimos 12 meses a coluna lombar (50%). A QV desses profissionais mostrou-se razoável, e, ao correlacionar a dor com os domínios da QV, notou-se correlação moderada e significativa entre ela e os domínios físico (p=0,010) e relações sociais (p=0,011). Não foram encontradas relações entre as características gerais dos participantes com a QV e a presença de dor.

**Conclusão:**

O aparecimento de sintomas musculoesqueléticos em profissionais de saúde da UTI pode repercutir na vida deles e interferir no seu aspecto social e no seu desempenho ocupacional.

## INTRODUCTION

Intensive care units (ICU) are hospital departments characterized by complex technologies and specialized staff to provide care to critically patients with recoverable conditions. Frequent occurrence of emergencies make this working environment hostile, stressful and emotionally demanding, this is to say, unhealthy for workers, in addition to the possibility of accidents in association with carelessness or lack of due training^1^^,^^2^^,^^3^.

ICU staff comprises physicians, nurses, physical therapists, pharmacists, nutritionists, psychologists and nursing technicians. All these workers are daily exposed to biological, physical, chemical and ergonomic hazards. Risk factors further include exhausting work schedule, exposure to infected materials, poor diet and inadequate furniture with the consequent postural risk. Low wages compel many such workers to seek a second job, whence their time for leisure activities and family is shortened, a fact that alienates them from their personal goals^4^^,^^5^^,^^6^^,^^7^.

The aforementioned factors might interfere with the state of health of ICU workers, manifesting e.g. as work-related musculoskeletal disorders (WMSD)-which include pain, fatigue, formication and feeling of heaviness. These problems intrude in the daily routine and are a frequent cause of sick leave^8^.

Work is directly related to quality of life (QoL) inasmuch as people spend most of their time at work, in addition its impact on well-being even on their free time^9^. A study performed in 2017 found that health care workers are the occupational group with the third highest level of stress and poorest QoL. To remind, QoL reflects the self-perception of people relative to their place in life, cultural environment, goals and expectations^10^.

Having more than one job and physical exhaustion derived from the job demands are some of the stressors with direct influence on ICU workers’ QoL. The reason is that difficult handling and the high dependency of patients pose a considerable overload to this population of workers^7^^,^^11^.

Given the small number of recent studies on this subject, the aims of the present study were to investigate musculoskeletal symptoms among ICU workers at a public hospital in Teresina, Piauí, Brazil, and analyze their correlation to QoL.

## METHODS

The present cross-sectional, quantitative, observational and analytical study was performed at the ICU of a public hospital in Teresina. We included 30 health care workers, from both sexes, out of a population of about 100 who agreed to participate and signed an informed consent form.

The present study was approved by the research ethics committee of State University of Piauí, ruling no. 1,938,852, Certificate of Presentation for Ethical Appraisal no. 64447617.0.0000.5209, in compliance with the National Health Council Resolution no. 466/12. We ensured full confidentiality and informed the participants as to possible risks and benefits of the present study. Questionnaires were administered by the investigators to the participants during their working hours.

Data collection was performed from April through September 2017 and involved administration of the version of the Nordic Musculoskeletal Questionnaire (NMQ) validated for the Portuguese language by Pinheiro et al.^12^^,^ which requires respondents to indicate musculoskeletal complaints on several body sites, and the World Health Organization Quality of Life scale (WHOQOL-BREF) validated for use in Brazil by Fleck et al.^13^ WHOQOL-BREF analyzes the main components of QoL for the previous 15 days. It comprises 26 items, being two for satisfaction with own QoL and 24 distributed across four domains (physical health, psychological, environment and social relationships).

We further administered a questionnaire to investigate the sample profile, including general information (age, sex, marital status and occupation), time in the profession, time in the job, working hours, number of jobs and breaks during the work shift. The data were entered on a Microsoft Excel 2010 spreadsheet. Statistical analysis was performed with software Statistical Package for the Social Sciences (SPSS®) version 21.0. The results were expressed as mean and standard deviation. Correlation was investigated by means of Spearman’s test and categorized as weak (0.00-0.39), moderate (0.40-0.69) or strong (0.70-1.00). The significance level was set to p<0.05.

## RESULTS

The sample comprised 30 ICU workers who agreed to participate; their sociodemographic and occupational data are described in Table 1. Most participants (70%) had one single job and 96.7% least one rest break during the work shift.

**Table 1.** Sociodemographic and occupational data, Teresina, Piauí, Brazil , 2019 (n=30)

Characteristics	N (%)	Mean±SD	Minimum	Maximum
Age (years)	30	42±13	23	70
Female sex	24 (80)	-	-	-
Marital status				
Married	15 (50)	-	-	-
Single	10 (33.3)	-	-	-
Divorced	3 (10)	-	-	-
Widowed	2 (6.7)	-	-	-
Years in the profession	-	15±14	0.58	40
Years in the job	-	10±11	0.16	35
Number of jobs	-	1±1	1	4
Weekly working hours	-	49±15	84	24
Occupation				
Nursing technician	11 (36.7)	-	-	-
Physical therapist	6 (20)	-	-	-
General services assistant	2 (6.7)	-	-	-
Operations assistant	1 (3.3)	-	-	-
Nurse	4 (13.3)	-	-	-
Psychologist	3 (10)	-	-	-
Speech therapist	1 (3.3)	-	-	-
Nutritionist	1 (3.3)	-	-	-
Patient transport	1 (3.3)	-	-	-

SD: standard deviation

We analyzed the frequency of pain, tingling and numbness complaints. The prevalence of pain in at least one body site was 86.7%. The knees were the most frequent location of complaints in the previous 7 days (23.3%) and the lower back in the previous 12 months (50%). NMQ evidenced that pain had implications for the participants’ lives, since it compelled them to seek medical care and disabled them for activities of daily living under some circumstances (Table 2).

**Table 2.** Frequency of pain and impact of musculoskeletal symptoms, Teresina, Piauí, Brazil , 2019 (n=30)

Pain frequency and impact	Shoulder	Neck	Chest	Elbow	Wrist/hand	Lower back	Hip	Knee	Foot
Pain (past 12 months)	8 26.7%	11 36.7%	13 43.3%	2 6.7%	7 23.3%	15 50%	2 6.7%	8 26.7%	4 13.3%
Pain (past 7 days)	3 10%	5 16.7%	5 16.7%	0 0%	1 3.3%	5 16.7%	4 13.3%	7 23.3%	5 16.7%
Interference with ADL	1 3.3%	2 6.7%	0 0%	0 0%	3 10%	2 6.7%	0 0%	0 0%	1 3.3%
Sought medical care	0 0%	0 0%	1 3.3	0 0%	3 10%	2 6.7%	1 3.3	0 0%	0 0%

ADL: activities of daily living

**Figure 1.**
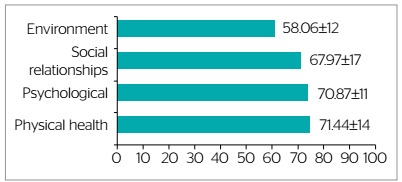
WHOQOL-BREF domain scores, Teresina, Piauí, Brazil, 2019 (n=30)

QoL was overall reasonable, since although the maximum score was not attained in any domain, the average score was over 50 (range: 0-100) (Figure 1).

**Table 3.** Correlation between pain and other variables of interest, Teresina, Piauí, Brazil , 2019 (n=30)

Correlation	Strength of correlation	P
Pain and physical health	0.461*	0.010**
Pain and psychological domain	0.309	0.097
Pain and social relationships	0.459*	0.011**
Pain and environment	0.285	0.127
Pain and age	0.176	0.353

P: Spearman’s test, p<0.05; *significant; **moderate correlation

We found significant moderate correlation between pain and WHOQOL domains physical health (p=0.010, rho=0.451) and social relationships (p=0.011, rho=0.459). None of the analyzed general characteristics (age, sex, time in the profession, time in the job, number of jobs, working hours) exhibited correlation with QoL or pain.

## DISCUSSION

Most participants reported pain in at least one body site, and their QoL was satisfactory, despite an association between pain and WHOQOL domains physical health and social relationships.

The sample profile agrees with that reported by Paschoa et al.^11^, since most participants had one single job, were mainly female, married or single. Upon analyzing nurses allocated to neonatal and pediatric ICU, Fogaça et al.^14^ reported a predominance of women and workers with more than 5 years in the profession, as also we found. According to Donoso^15^ such strong presence of women in health care professions is historically related to the division of labor within the traditional family structure, according to which women are responsible for the care of children, older and sick relatives. However, together with their increasing social mobility, women have achieved autonomy and gained access to new environments, as e.g. the labor market.

Our results for pain agree with reports in the literature. For instance, Ribeiro et al.^16^ found pain complaints among more than half of the participants in their study (83.4%) mainly involving the lower back and the legs. Similarly, Gurgueira et al.^17^ reported that the lower back, knees and shoulders were the most frequently affected body sites in the previous 12 months. Magnano et al.^18^ observed that 96.3% of their sample described musculoskeletal symptoms in some body site, and Smith et al.^19^ pain among 70% of the participants in their study. In these two studies the lower back, neck, shoulder and knees were the most frequently involved body sites. All the participants in Martins’^20^ study reported some complaint in the previous 12 months, particularly in the upper or lower back. ICU workers spend most of their time at work standing, walk long distances and handle heavy weights, being compelled to adopt awkward postures and to perform strong physical effort. All these factors might be associated with the reported high rates of lower back and knee pain.

In our and the aforementioned studies, while symptoms were similar for all occupational categories most participants were nurses and nursing technicians. In a study performed in South Korea by Kee and Sao^21^, 56.8% of nursing professionals reported musculoskeletal symptoms lasting at least one week along the previous 12 months. These findings are a cause of much concern, since pain has impact on everyday life and interferes with daily activities, in addition to causing job dissatisfaction, therefore, with influence not only on QoL but also on the operation of staffs.

Although we did not find low scores on any WHOQOL domain, it is influenced by musculoskeletal symptom. For instance, Bonzini et al.^22^ analyzed the relationship between these symptoms and job tasks and found association with psychological factors such as low mood, job dissatisfaction, occupational stress and somatization.

In agreement with our findings, Paschoa et al.^11^ reported that the lowest score corresponded to WHOQOL domain environment, which analyzes the physical environment, safety, financial resources, participation and opportunities for leisure activities, the home environment, transport and access to health. The reason might be related to the occupational hazards inherent to the ICU environment. However, in their study the highest score corresponded to domain social relationships, while in ours to physical health, followed by the psychological domain. Also in their study conducted in Chile, Andrades and Valenzuela^23^ administered WHOQOL-BREF and found that social relationships was the domain with the highest score and physical health that with the lowest score, thus once again diverging from our results. A possible explanation is that the analyzed population spends too much time at work and thus little is available for leisure activities.

We found significant moderate correlation between pain and WHOQOL domains physical health and social relationships. These findings agree with those reported by Meira, Mascarenhas and Sampaio Miranda^24^ for a sample of community health agents, to wit, significant correlation between QoL and musculoskeletal pain (p<0.001). Similarly, Mergener et al.^25^ tested associations between prevalence of WMSD and QoL among bank employees. The results indicated high prevalence of WMSD, with weak correlation between QoL psychological, social and environment domains, but moderate negative correlation with domain physical health (rho=-0.411, p<0.001).

We did not find any relationship between any of the analyzed general characteristics of the participants and WHOQOL scores. Differently, Paschoa et al.^11^ reported significant (p<0.05) weak correlation with QoL domains physical health, psychological and social relationships.

The present study has several limitations derived from the small sample size and the fact it was performed at a single ICU. In addition, the element of subjectivity implicit in the responses on the administered questionnaire might have behaved as a source of bias in statistical analysis. Nevertheless, we emphasize the relevance of our study, as well as the need for further investigation of this subject with larger samples and more questionnaires.

## CONCLUSION

Most participants reported musculoskeletal symptoms mainly involving the knees and lower back, which may interfere with their social life, performance at work, and therefore also with their QoL. Participatory health promotion and workplace well-being initiatives are needed to improve the quality of life of ICU workers.
